# Takotsubo cardiomyopathy in a 68-year old Russian female

**DOI:** 10.1186/1757-1626-1-64

**Published:** 2008-07-28

**Authors:** Suriya Jayawardena, Danushan Sooriabalan, Olga Burzyantseva, Selvarathnam Sinnapunayagm

**Affiliations:** 1 Dept of Cardiology, Coney Island Hospital, 2601 Ocean Parkway Brooklyn, NY 11235, USA; 2 Dept of Medicine, Coney Island Hospital, 2601 Ocean Parkway Brooklyn, NY 11235, USA

## Abstract

**Introduction:**

Takotsubo cardiomyopathy also known as transient left ventricular apical ballooning, stress-induced cardiomyopathy can present with retrosternal chest pain with EKG changes that can mimic a myocardial infraction.

**Case Presentation:**

We present a 68 female with sudden onset retrosternal squeezing chest pain with positive cardiac enzymes and EKG changes suggestive of acute ST-elevation myocardial infraction. Patient was thrombolysed and cardiac cauterization done later showed normal coronaries with ballooning of the left ventricle apex.

**Conclusion:**

Takotsubo cardiomyopathy is a very rare disease entity yet can present to the emergency room as acute myocardial infraction.

## Introduction

Takotsubo cardiomyopathy also known as transient left ventricular apical ballooning, stress-induced cardiomyopathy, broken heart syndrome, was first described in Japan fifteen years ago by Dote and his colleagues. The word "takotsubo" is the Japanese word for an octopus fishing trap with a round bottom and narrow neck which is used to describe the left ventricular apical ballooning with compensatory hyperkinesis of the basal walls seen on left ventriculogram during systole. This reversible form of cardiomyopathy which mostly affects postmenopausal women has a presentation very similar to an acute myocardial infarction, except for the fact that cardiac catheterization reveals normal coronary arteries. Left ventricular dysfunction will normally improve rapidly within a few weeks and with no treatment despite the acute left sided heart failure and hemodynamic compromise seen in many patients. Although a clear etiology and pathogenesis are still unknown, numerous studies point to a catecholamine-induced coronary vasospasm brought upon by an acute medical illness or intense emotional or physical stress causing myocardial stunning. The mortality rate and risk of recurrence are very low.

## Case presentation

A 68-year old Russian female was brought the emergency department by paramedics with the complain of sudden onset left sided retrosternal squeezing chest pain radiating to the left shoulder and the back with a intensity of 7/10 associated with shortness of breath. The pain began while she was mopping the floor. It was the first time patient had experienced such pain. She has no significant past medical or a family history of coronary artery disease and was not on regular medication except for occasional over the counter pain medications.

**Figure 1:**
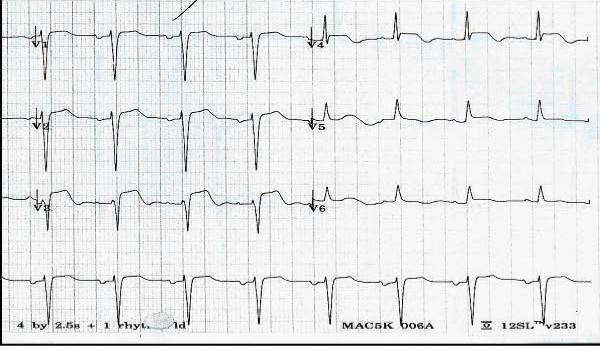
EKG on admission showing ST-elevation in leads V_3_–V_5_and T-wave inversion in leads V_4_–V_5_.

Upon arrival she was found to have blood pressure of 80/40 mm Hg, heart rate of 56 beat/min, respiratory rate of 24/min and a temperature of 98°F, oxygen saturation of 98% on room air. On examination S1 S2 was regular no murmurs appreciated and basal rales were noticed and the rest of the physical examination was normal The Electrocardiogram (EKG) done on arrival showed ST-elevation in leads V_3_–V_5 _with T-wave inversions in leads V_4_–V_5 _(Figure 1) Creatine phosphokinase (CPK) with mass, index and Troponin-I levels were only mildly elevated with a peak CPK of 415 U/L (10–225), CKMB mass of 20 ng/ml (0–10), CK index of 4.8 (0.0–3.0) and a peak Troponin I of 0.6 microg/L (< = 0.1). An initial diagnosis of anterior wall ST elevation myocardial infraction was made; aspirin 162 mg orally with morphine 2 mg and phenergan 12.5 mg intravenous was given. Beta blockers were held as the patient was hypotensive and bradycardic, she was given a fluid challenge with 1 liter of normal saline over half an hour. The echocardiogram (Echo) done in the emergency room revealed hypokinesia of the anterior wall, septum, and the apex, left ventricular ejection fraction of 35–40%, poorly visualized left ventricular outflow tract (LVOT) and mitral regurgitation of unknown severity.

With the ongoing chest pain, EKG changes, positive cardiac enzymes and the echocardiographic findings patient was thrombolysed with 2 doses of Retavase, 30 minutes apart followed by intravenous heparin and transferred to the coronary care unit. As there was no improvement in blood pressure (70/40 mmHg) with a fluid challenge and after 3 liters of normal saline infusion and worsening Shortness of breath, Dopamine infusion was started to improve the blood pressure. Three hours later as there was no improvement of the blood pressure, worsening pulmonary edema and drop in oxygen saturation on nasal cannula prompted the decision to intubate and place an intra-aortic balloon pump.

**Figure 2:**
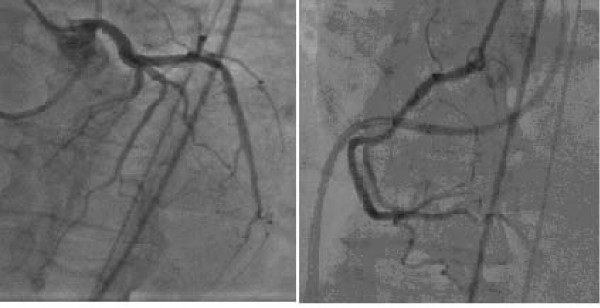
Coronary angiography showing no stenotic lesions of the left coronary artery, circumflex artery (A), and right coronary artery (B).

**Figure 3:**
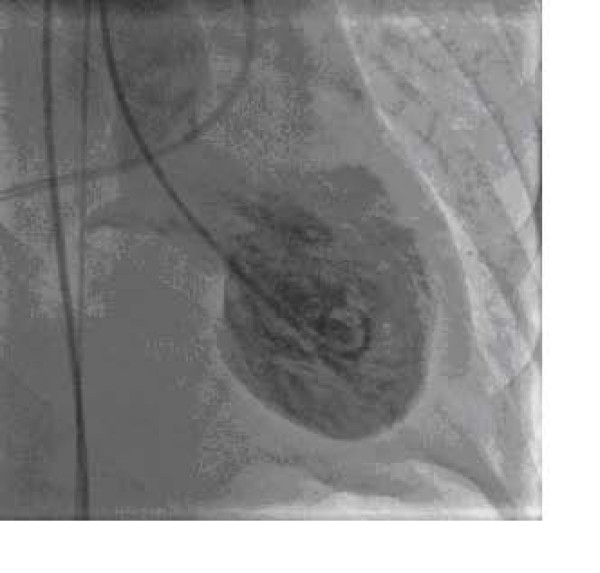
**Left ventriculography during end-systole showing systolic dysfunction of the left ventricular apex and mid-ventricle, with hyperkinesis of the basal left ventricular segments (A). **This finding on left ventriculography resembles the look of a Japanese takotsubo pot which was traditionally used for catching octopus.

She was subsequently transferred to hospital with invasive cardiac catheterization facilities for emergency cardiac catheterization Coronary angiography revealed non obstructive coronary arteries (Figure 2). Left ventriculogram showed systolic dysfunction of the left ventricular apex and mid-ventricle, with hyperkinesis of the basal left ventricular segments and LVOT blood flow gradient of 58 mmHg and a 2+ mitral regurgitation (Figure 3).

Over the next few days, patient's blood pressure improved and she was extubated and the balloon pump was removed. Patient was asymptomatic and physical examination was normal. Her EKG returned to normal and repeat Echo was entirely normal with no wall motion abnormalities, no LVOT obstruction and no mitral regurgitation and an ejection fraction of 60%. Patient was discharged on Aspirin 81 mg daily and Metoprolol 25 mg twice a day. Patient was followed up for a period of one year there was no recurrence of her clinical symptoms and the repeat echocardiogram as well as stress echo was normal.

## Discussion

Based on the findings of transient left ventricular dysfunction involving the middle and apex portion of the left ventricle with no stenotic lesions on coronary angiography, we believe that our patient presented with Takotsubo's cardiomyopathy. This form of cardiomyopathy was first reported in Japan by Dote and his colleagues [1]. The name "Takotsubo" comes from the Japanese word for an octopus trap (takotsubo) that has a similar shape to the left ventricular apical ballooning seen on left ventriculography [2-5]. Although most cases were initially found in Japan, more recent reports show that this form of cardiomyopathy has been reported in western countries [6]. In the literature, this disease seems to account for about 1% of all presumed acute myocardial infarctions [1,7].

Our patient was a 68-year old, postmenopausal woman. Studies show that stress-induced cardiomyopathy is more common in women than men [3,4,8]. In a systematic review, postmenopausal women accounted for 82 to 100 percent of cases, with a mean age of 62 to 74 years [3]. An explanation for the strong predominance in the female population is unclear. Some studies point to alterations of endothelial function in response to reduced estrogen levels in postmenopausal women [9].

Most cases have a history of preceding physical or emotional stress [4,6]. Our patient was mopping the floor before she noticed her symptoms. Chest pain is the most frequent symptom of presentation [5]. Although this cardiomyopathy does mimic that of an acute myocardial infarction, a coronary angiogram will show no stenotic lesions. Based on one study, patients with cardiomyopathy were older than patients with anterior acute myocardial infarction (75 +/- 10 VS 57 +/- 16 years, p = 0.002) [10]. The same study found that T-wave inversions were seen in 4 patients (31%) with Takotsubo cardiomyopathy, but in none of the patients with anterior myocardial infarction [10]. In addition, ST-segment elevation was seen primarily in leads V_1–3 _in patients with anterior acute myocardial infarction, whereas it was elevated primarily in leads V_4–6 _in patients with Takotsubo cardiomyopathy [10]. One postulate for this EKG finding is that Takotsubo cardiomyopathy is confined to the apical wall, and therefore the ST-segment elevations are greater in leads V_4–6 _than in leads V_1–3._[10]. Our patient had T-wave inversions in leads V_4–5 _and ST-elevations in leads V_3–5_.

In addition to using EKG findings to distinguish Takotsubo cardiomyopathy from an acute myocardial infarction, cardiac enzymes can also be used. CPK and Troponin levels often rise slightly in Takotsubo cardiomyopathy compared with acute myocardial infarction [4,7,10]. This finding was also seen in our patient who had only minimally elevated CK-MB and Troponin-I.

Our patient also had LVOT obstruction, mitral regurgitation of unknown severity. Isolated cases of left ventricular mural thrombus formation have been reported [11]. We inserted an intra-aortic balloon pump and administered dopamine for the hypotension. In one study up to 18% of cases demonstrated LVOT obstruction in the acute phase and disappears thereafter [5]. LVOT obstruction induced by left ventricular basal hyperkinesis can contribute to the development of shock and cause severe mitral regurgitation or the murmur of hypertrophic cardiomyopathy [12]. Our patient was administered dopamine for her hypotension; however the recommended approach for patients with LVOT obstruction consists of beta blockers, phenylephrine, and fluid resuscitation in the absence of pulmonary congestion [3,12]. Hypotension associated with LVOT obstruction should not be treated with inotropic agents such as dobutamine and dopamine, which can worsen the degree of obstruction [8,12].

The etiology of Takotsubo cardiomyopathy is still unknown. Several mechanisms have been proposed, including multivessel epicardial spasm, myocardial dysfunction mediated through catecholamine-induced damage, microvascular coronary spasm or dysfunction, and neurogenically mediated myocardial stunning [3]. Multivessel coronary vasospasm, which would probably be required to produce the diffuse wall-motion abnormalities seen with this syndrome, has been present in 13 of 73 patients tested in one study [3]. Some researchers suggest a catecholamine-mediated stunning of the myocardium, which is provoked by emotional or physiologic stress [13]. Patients presenting with this syndrome seem to have abnormalities of cardiac sympathetic innervations with evidence of hyperactivity at the cardiac apex [14,15]. One group of researchers have recently shown transient left ventricular hypocontraction via excessive activation of cardiac adrenoreceptors in the animal model [16]. We did not check the catecholamine levels of our patient, thus cannot comment on whether increased catecholamine levels played some role in inducing her cardiomyopathy.

Treatment is variable and includes pharmacological and mechanical hemodynamic support as needed [4]. After diagnostic arteriography, supportive medical management includes administration of B-blockers, angiotensin-converting enzyme inhibitors (in patients without an intra-cavitatory gradient), aspirin, and intravenous diuretics as needed [3]. Hypotension must be evaluated for a dynamic intraventricular pressure gradient in the left ventricular cavity and left ventricular outflow tract [3]. Short-term anticoagulation should be considered in most patients to prevent left ventricular mural thrombus formation.

Recurrences are very unusual [6]. Patients who survive the acute episode typically recover normal ventricular function within one to four weeks [4,6,8]. Our patient's left ventricular ejection fraction was 60% on the sixth day after her symptoms began. This unique form of cardiomyopathy should be considered in the differential diagnosis of any patient presenting with an apparent acute coronary syndrome in the absence of obstructive atherothrombosis [3].

## List of Abbreviations

EKG: Electrocardiogram; Echo: Echocardiogram; CPK: Creatinine phosphokinase; CK: Creatinine Kinase; LOVT: Left Ventricular Outflow Tract.

## Competing interests

The above mentioned authors declare that they have no competing interests. The above case report was written at Coney Island Hospital. The above mentioned authors have no affiliation to any other institute other than Coney Island Hospital.

## Authors' contributions

SJ, DS and OB treated the patent and were responsible for writing the paper and looking up the back ground references, SS was responsible for over all coordination and final proof reading. All the above mentioned authors read and approved the final manuscript.

## Consent

A written informed consent was obtained from the patient for publication of this case report and accompanying images. A copy of the written consent will be made available on request.
